# Obesity, Knowledge, and Perceived Risk: Insights from the ObeCare Project Across Italian Territorial Pharmacies

**DOI:** 10.3390/healthcare13212793

**Published:** 2025-11-04

**Authors:** Francesco Ferro Russo, Federica Faccitondo, Vladimiro Grieco, Eugenio Leopardi, Stefania Agrimi, Gian Maria Rossi, Anna Cantarutti, Benedetta Canova, Riccardo Boracchini, Paolo Levantino

**Affiliations:** 1 Fenagifar (National Federation of Associations of Young Pharmacists), Via dei Tizii 10, 00185 Rome, Italy; francescoferrorusso@gmail.com (F.F.R.); f.faccitondo@icloud.com (F.F.); vladimiro.grieco@gmail.com (V.G.); stefania.agrimi@gmail.com (S.A.); gianmaria.rossi.25@gmail.com (G.M.R.); 2 Utifar (Italian Technical Union of Pharmacists), Piazza Duca d’Aosta 14, 20124 Milan, Italy; eugenioleopardi@gmail.com; 3 Department of Statistics and Quantitative Methods, Division of Biostatistics, Epidemiology and Public Health, University of Milan-Bicocca, Piazza dell’Ateneo Nuovo 1, 20126 Milan, Italy; anna.cantarutti@unimib.it (A.C.); benedetta.canova@unimib.it (B.C.); riccardo.boracchini@unimib.it (R.B.)

**Keywords:** obesity, lifestyle behaviors, risk perception, pharmacist-led intervention, public health education

## Abstract

**Background:** Obesity is a growing public health concern in Italy, with prevalence reaching 21.6% in 2022, particularly among the elderly. To address this issue, the ObeCare project was implemented to promote obesity prevention and awareness through community pharmacies. This study aimed to evaluate obesity-related knowledge, lifestyle behaviors, and risk perception among participants engaged in the ObeCare project. **Methods:** A survey was administered to individuals >18 years old across Italian pharmacies involved in the ObeCare initiative by trained pharmacists. A validated questionnaire assessed obesity-related knowledge, risk perception, lifestyle behaviors, demographics, clinical conditions, and Body Mass Index (BMI). A multinomial logistic regression was implemented to identify predictors of overweight and obesity. **Results:** Obesity was more prevalent among men, older adults, and those with multiple comorbidities. Healthier lifestyle and greater health knowledge were significantly associated with having a normal BMI, whereas low lifestyle scores (OR 13; 95% CI 2.96–57.85) and the presence of multiple comorbidities (OR 14.79; 95% CI 8.00–27.36) were strong predictors of obesity. Individuals with obesity exhibited lower knowledge (OR 1.55; 95% CI 1.02–2.37) and risk perception. **Conclusions:** This study highlights the significance of lifestyle habits, knowledge, and risk perception, which will be helpful in the prevention and management of obesity. These findings support community-based education and targeted interventions, especially for high-risk groups such as men, retirees, and residents of Southern Italy.

## 1. Introduction

Obesity is a chronic, relapsing disease marked by excessive adiposity with or without abnormal distribution or function of adipose tissue arising from complex interactions among genetic, biological, environmental, behavioral, and psychosocial factors [1,2]. Obesity is also recognized as a state of chronic low-grade inflammation, which contributes to its pathophysiological complications [3]. Strongly linked to increased morbidity and mortality, obesity contributes to the onset and worsening of numerous chronic inflammatory and non-communicable diseases (e.g., type 2 diabetes, cardiovascular disease, certain cancers), placing a significant burden on healthcare systems and quality of life [4].

Obesity management requires a multidisciplinary approach involving medicine, nutrition, and psychology. Treatment must be tailored to the patient’s clinical condition and comorbidities, with sustained, personalized care [5,6]. In Italy, following the international guidelines, the treatment of obesity follows a stepwise approach: it begins with lifestyle behavioral interventions, followed by pharmacological therapy, and, in severe or refractory cases, by bariatric surgery [7]. While lifestyle changes, diet, and physical activity are key, long-term support is often needed due to biological, social, and behavioral barriers [8].

In Italy, 21.6% of adults are obese, reflecting a rising trend driven by lifestyle changes such as increased sedentary behavior and calorie-dense diets. From 1980 to 2013, overweight and obesity rose by 27.5% in adults and 47.1% in children [9]. The economic burden is substantial, with obesity costing EUR 13.34 billion annually, EUR 7.89 billion in direct healthcare, and EUR 5.45 billion in productivity losses. The issue is more severe in older adults, with 63.6% of those aged 65–74 overweight, highlighting the need for age-specific interventions [10]. Notably, according to World Health Organization (WHO) data, Italy still shows one of the lowest obesity prevalence rates in Europe when compared with many European countries (i.e., Croatia (35.7%), Greece (33.7%), Hungary (36.4%), United Kingdom (28.7%), Romania (38.2%)) underscoring its potential as a case study for broader regional strategies [11].

In recent years, community pharmacies have assumed an increasing role in public health, starting to provide health promotion and disease prevention. This evolution has been accelerated by regulatory changes during the COVID-19 pandemic, making pharmacies essential access points for information, medication delivery, and preventive services like vaccinations and health consultations [12]. In this context, the ObeCare project (promoted by Fenagifar in collaboration with Utifar) was designed to engage territorial pharmacies in obesity prevention and public education. Pharmacists delivered health content through digital and printed media and provided personalized in-pharmacy counseling to promote healthier behaviors and greater awareness of obesity-related risks. The educational materials provided to patients include information on the causes of obesity, the health risks associated with the condition, recommendations for diet and physical activity, and practical strategies for stress reduction. They also offer guidance on adopting sustainable lifestyle changes that can help prevent or manage obesity. Pharmacists can tailor their counseling to each patient’s age and weight, offering support with both professional expertise and empathy. When appropriate, they can also recommend regular monitoring of BMI, waist circumference, blood glucose, and lipid profile at the pharmacy.

This study analyzes data from the ObeCare initiative to (i) assess public awareness of obesity-related health risks, (ii) examine associations between knowledge, risk perception, and health behaviors, and (iii) generate evidence to inform scalable prevention strategies in healthcare and community settings.

## 2. Materials and Methods

### 2.1. Study Design and Setting

The ObeCare project was implemented through a network of territorial pharmacies distributed across Italy during January and February 2025, with representation from the North (35%), Center (12%), and South and Islands (85%). To ensure standardization in participant recruitment and data collection, all participating pharmacists underwent structured training prior to the initiation of survey administration. A cross-sectional study design was implemented. The training included an online webinar covering the ObeCare project, the definition, causes, and consequences of obesity, as well as the biological aspects of the microbiome and bacterial composition in individuals with obesity. Additionally, the webinar addressed the pathophysiological mechanisms linking obesity and hypertension and highlighted the benefits of engaging in regular physical activity.

### 2.2. Participants

Eligible participants included individuals aged 18 years or older who were not classified as underweight (Body Mass Index < 18.5) and who did not have a diagnosis of neurological disorders. All participants provided informed consent prior to participation. The survey was offered consecutively to all eligible individuals presenting at the participating pharmacies during the study period.

### 2.3. Survey Instrument

The questionnaire used in this study (Supplementary Materials—Questionnaire) was designed to assess obesity-related knowledge, lifestyle habits, risk perception, and motivational factors influencing health-related behaviors. The instrument was developed by translating into Italian, integrating, and adapting content from two internationally validated tools (i.e., Portuguese Obesity Knowledge Questionnaire (POKQ) [13] and Health Promoting Lifestyle Profile (HPLP) [14]).

To enhance cultural and contextual relevance, both instruments were modified by excluding non-applicable sections (e.g., items related to pharmacological treatment), rephrasing selected items for clarity, and adding culturally specific questions relevant to the Italian population. A Likert scale format was employed throughout the questionnaire to facilitate nuanced measurement of participant perceptions, attitudes, and behaviors.

Following completion of the questionnaire, participants received a personalized counseling session based on their questionnaire responses, aimed at promoting long-term behavioral change through empathic communication, practical guidance, and motivational support in areas such as nutrition, physical activity, and stress management.

The questionnaire was administered by pharmacists, who recorded participants’ responses using a Typeorm platform. The data were collected anonymously to ensure participant confidentiality.

### 2.4. Variables and Indicators

#### 2.4.1. Demographic and Clinical Variables

Demographic and clinical characteristics were collected, including sex, age, height, weight, and the presence of chronic conditions (e.g., diabetes, hypertension, cardiovascular disease, respiratory disorders, thyroid disease, gastrointestinal disorders, rheumatologic, neurologic, autoimmune, oncological, depressive and anxiety disorders, renal insufficiency, or other pathologies). In this work, we use the term *sex* to refer to the biological attribute.

Geographic location was determined based on the name and city of the pharmacy and was further classified using Nielsen areas (i.e., Area 1: Piedmont, Aosta Valley, Liguria, Lombardy; Area 2: Trentino-Alto Adige, Veneto, Friuli-Venezia Giulia, Emilia-Romagna; Area 3: Tuscany Umbria, the Marches, Lazio, Sardinia; and Area 4: Abruzzo, Molise, Puglia, Campania, Basilicata, Calabria, Sicily).

Body Mass Index (BMI) was calculated as weight in kilograms divided by height in meters squared (kg/m^2^). Based on WHO criteria, participants were categorized as normal weight (18.5 ≤ BMI < 25), overweight (25 ≤ BMI < 30), and obese (BMI ≥ 30) [15]. Participants were then classified into three groups: no comorbidities, one comorbidity, or more than one comorbidity.

#### 2.4.2. Questionnaire Domains

The questionnaire assessed three primary domains:

(a) Obesity-related knowledge was evaluated using 7 items (Q1, Q2, Q4–Q8) covering topics such as obesity prevalence in Italy, risk factors, and the definition of BMI. Correct answers were scored as 1 and incorrect answers as 0. The total score was rescaled to a 1–5 scale using a standard linear transformation $New\;Score=\frac{Old\;Score-Min_{old}}{Max_{old}-Min_{old}}\;\times \left(Max_{new}-Min_{new}\right)+Min_{new}$;

(b) Risk perception: perceived health risks associated with obesity were assessed using 6 items (Q3, Q9–Q13) and rated on a 5-point Likert scale. The average score across items was calculated, with higher values indicating a greater perception of obesity-related health risks;
(c) Lifestyle behaviors were assessed through 9 items (Q14–Q22), also rated on a 5-point Likert scale. Items addressed diet, physical activity, sleep quality, stress management, and health check-up frequency. The mean score was used as a composite indicator of overall lifestyle quality.

Scores within each domain were categorized to facilitate interpretation. For the Knowledge and Lifestyle indicators, four categories were defined based on the rescaled 1–5 score (low: 1–<2, medium-low: 2–<3; medium-high: 3–<4; high, 4–5). For the Risk Perception indicator, a three-level classification was applied (low: 1–<3, medium: 3–<4, and high: 4–5).

### 2.5. Statistical Analysis

Sociodemographic and clinical characteristics of the respondents were summarized using descriptive statistics. Categorical variables were presented as frequencies and percentages, while continuous variables were described using medians and interquartile ranges (IQRs), due to non-normal distribution. BMI, obesity knowledge, and risk perception scores were analyzed both overall and stratified by sex, age group, Nielsen area, lifestyle habits, and occupational status, in order to identify potential group differences.

A multinomial logistic regression model was used to identify BMI class predictors, providing Odds Ratios (ORs) and 95% Confidence Intervals (95% CIs). The model has been adjusted by sex, age at the survey, Nielsen area, occupational status, number of comorbidities, knowledge score, and lifestyle score. The perception score was excluded from the model as it was not deemed predictive of BMI category.

We followed the Checklist for Reporting Of Survey Studies (CROSS). All statistical analyses were performed using SAS software, version 9.4 (SAS Institute, 17 Inc., Cary, NC, USA) and R Statistical Software version 3.6.1 (R Foundation for Statistical Computing, Vienna, Austria).

## 3. Results

### 3.1. Survey Responses and Participants’ Characteristics

A total of 1081 questionnaires were collected, out of which 831 (76.87%) had complete information and were included in the analysis (Figure S1—Panel A). Notably, 51% of the respondents were from Area 4 (Figure S1—Panel B). Table 1 presents the sociodemographic and clinical characteristics of the respondents. The majority were female (60%), with a median age of 48 years (IQR, 35–61 years), and employed (69%). Hypertension was reported by 20% of participants, followed by thyroid disorders (12.7%), diabetes (9.8%), gastrointestinal conditions (9.4%), obesity (8.9%), and cardiovascular diseases (8.5%). A majority of respondents (61.1%) demonstrated healthy lifestyle patterns, while 38.8% showed suboptimal lifestyle behaviors.

### 3.2. BMI

Overall, 353 respondents (41%) had a normal weight, 288 (34%) were overweight, and 202 (24%) were classified as obese. Men had significantly higher rates of both overweight (45.85% vs. 26.91%) and obesity (26.15% vs. 22.49%) compared to women (*p* < 0.001). Individuals with obesity were older, with a median age of 56 years, compared to 52 years among overweight participants and 40 years among those with normal weight (*p* < 0.001). Obesity was more prevalent among retired or unemployed individuals than in other occupational categories (*p* < 0.001). Furthermore, higher rates of obesity were observed in participants from Area 1 and Area 4 (*p* < 0.001). Individuals with obesity had significantly higher prevalence of diabetes, hypertension, and cardiovascular diseases compared to those with normal weight or overweight (*p* < 0.001). They also showed increased rates of chronic respiratory diseases, gastrointestinal disorders, and rheumatic conditions (*p* < 0.04). Among participants without any comorbidities, 57% were of normal weight (*p* < 0.001). Respondents with normal weight reported significantly healthier lifestyle patterns compared to those with obesity, who were more frequently classified in the low or medium-low lifestyle categories (*p* < 0.001) (Table 1).

### 3.3. Obesity Knowledge and Perception

Our findings on obesity-related knowledge aligned with the previously reported results, showing significantly higher levels among women compared to men (*p* = 0.01), among employed individuals and students compared to other occupational groups (*p* < 0.01), in participants without comorbidities (*p* < 0.001), and among those with healthy lifestyle patterns (*p* < 0.001) (Table S1).

However, overall risk perception was high among respondents, with significantly higher levels observed in younger individuals (*p* = 0.018). Additionally, a clear increasing trend in risk perception was found in association with healthier lifestyle patterns (*p* < 0.001) (Table S1).

### 3.4. Models

#### 3.4.1. Overweight Compared to Normal Weight

**Figure 1:**
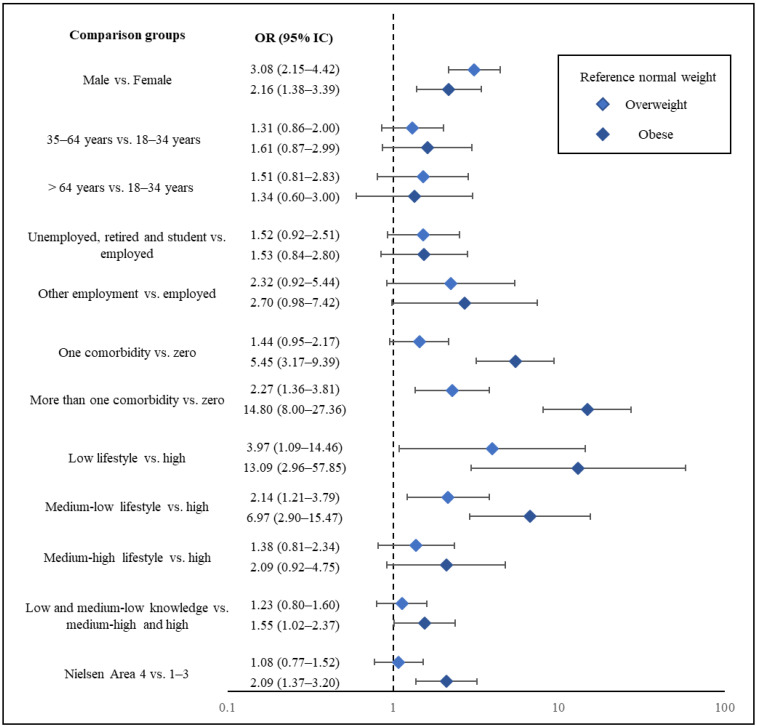
Forest plot representing Odds Ratios (ORs) and corresponding 95% Confidence Intervals (CIs) for overweight and subjects with obesity, using individuals with normal weight as the reference group. ORs and 95% CI are derived from a multinomial logistic regression model.

Men had a significantly higher odds of being overweight compared to women (OR = 3.08; 95% CI: 2.15–4.42). The presence of at least one comorbidity seemed to be associated with an increased odds of overweight (OR = 1.43; 95% CI: 0.95–2.17), which became significant in individuals with two or more comorbidities (OR = 2.27; 95% CI: 1.35–3.81). An inverse association was observed between lifestyle behavior and overweight odds: participants with poorer lifestyle habits showed progressively higher odds of being overweight (low: OR = 3.96; 95% CI: 1.08–14.46; middle-low: OR = 2.14; 95% CI: 1.21–3.78; middle-high: OR = 1.38; 95% CI: 0.81–2.34). Additionally, the odds of being overweight appeared to increase slightly with age (OR range: 1.3 to 1.5) and were higher among individuals with precarious employment status, including those who were inactive (OR = 1.52, 95% CI: 0.92–2.51) or had unspecified occupations (OR = 2.32, 95% CI: 0.92–5.44) (Figure 1).

#### 3.4.2. Obese Compared to Normal Weight

Males had a significantly greater odds of being overweight compared to females (OR = 2.16; 95% CI: 1.38–3.39). Having at least one comorbidity substantially increased the odds of being overweight (OR = 5.45; 95% CI: 3.17–9.39), with an even greater odds observed among those reporting two or more comorbid conditions (OR = 14.79; 95% CI: 8.00–27.36). A strong inverse relationship was found between lifestyle quality and overweight odds: individuals with lower lifestyle scores had markedly higher odds of being overweight (low: OR = 13.00; 95% CI: 2.96–57.85; middle-low: OR = 6.69; 95% CI: 2.90–15.47; middle-high: OR = 2.08; 95% CI: 0.92–4.75). Interestingly, overweight odds appeared to decline modestly with increasing age (OR range: 1.6 to 1.3). Moreover, those with precarious employment—particularly inactive individuals (OR = 1.53, 95% CI: 0.84–2.80) and those with undefined occupations (OR = 2.70, 95% CI: 0.98–7.42)—were more likely to be overweight (Figure 1).

## 4. Discussion

In this study, based on 831 completed surveys, we explored the sociodemographic and clinical characteristics of participants in relation to BMI classification, as well as their levels of obesity-related knowledge and risk perception. Our results showed that men were more likely to be overweight or obese than women and that healthier lifestyle patterns were strongly associated with normal weight status and lower obesity rates. Obesity was frequently observed in individuals with chronic conditions such as diabetes, hypertension, and cardiovascular diseases. While the overall perception of obesity as a health risk was high, it was notably lower among participants with obesity. Similarly, obesity-related knowledge was higher among individuals with normal weight and decreased progressively with increasing BMI. Multivariable models further confirmed that males, inactive individuals, and those with multiple comorbidities had an increased odds of overweight or obesity.

Consistent with prior research, men were more likely to be overweight or obese than women (OR = 3.08 and 2.16), reflecting global patterns of sex differences in BMI and health behaviors [16]. Although women showed slightly lower knowledge levels, they had better weight status and a higher perception of obesity as a health risk, in line with findings from the WHO European Region [17]. Age-related trends also supported previous evidence: middle-aged adults were more prone to obesity, while younger individuals, particularly students, were more often normal weight and demonstrated higher knowledge. These results align with Malik et al. (2013), who linked aging with lifestyle deterioration and increased BMI [18]. Geographically, obesity rates were highest in Nielsen Area 4 (Southern Italy), reflecting regional disparities in socioeconomic status and cultural dietary patterns, which have been proven to influence lifestyle and obesity risk, highlighting the need for targeted interventions [19]. Finally, we observed a clear gradient of odds with increasing comorbidities, reinforcing the bidirectional relationship between obesity and chronic disease, particularly diabetes and cardiovascular conditions, as widely documented [20].

The inverse relationship between lifestyle quality and BMI was clear: 60% of individuals with a healthy lifestyle were normal weight, compared to just 19.2% among those with poor habits. This aligns with growing evidence supporting lifestyle interventions in obesity prevention [21]. Our models confirmed this gradient, showing a sharp rise in obesity odds with poorer lifestyle levels (OR = 13.09 for low lifestyle). A 2025 meta-analysis similarly reported a 43% lower risk of metabolic syndrome among individuals with the healthiest lifestyle patterns (OR ≈ 0.57) [22]. Comparable trends have been observed in adolescents, linking higher lifestyle risk scores to increased odds of overweight and obesity [23].

Knowledge and perception of obesity emerged as a potential determinant of weight outcomes. Individuals with obesity showed lower knowledge and risk perception than individuals with normal weight, suggesting lower awareness that may contribute to unhealthy behaviors and higher BMI values, which reduce perceived control or urgency as shown by Zalewska et al. and Robinson et al. in 2022 [24,25]. The crucial role of having higher perception and higher knowledge underscores the potential role of education in shaping healthier attitudes and behaviors, a core principle in health promotion strategies [26].

This study provides an integrative analysis of BMI status in relation to lifestyle behaviors, chronic diseases, sociodemographic variables, disease knowledge, and the way disease is perceived as a risk factor among a large and diverse population sample. By combining all the information gathered from the questionnaire, such as health status, anthropometric measures, and self-awareness variables, it offers a comprehensive perspective on the multifactorial nature of obesity. The strong associations between lifestyle and obesity, as well as between knowledge and perception and health outcomes, highlight the need for the implementation of public health strategies focused on education, behavioral change, and regional adaptation. In addition, this study reveals critical gaps in knowledge in at-risk groups (e.g., males, retirees, Area 4 individuals), which should be considered key targets for intervention by the local health authority. Additionally, a subset of participants was asked to complete a second survey after receiving informational material about the importance of the factors examined in the initial questionnaire. This follow-up survey assessed the usefulness, clarity, and relevance of the materials, as well as participants’ willingness and motivation to make lifestyle changes aimed at improving quality of life and achieving healthier habits. More than 55% of respondents rated the materials as useful and clear. While only 27% reported being highly willing to make lifestyle changes, 55% indicated they would make at least some changes. Regarding motivation, 34% of respondents stated that they felt strongly motivated, while 35% reported being moderately motivated (Supplementary Materials—Questionnaire Feedback, Figure S2). These results emphasize the importance of not only providing information but also creating structured, accessible educational programs that can enhance motivation and foster sustained behavior change. Improving health outcomes requires supporting individuals at all levels of readiness, particularly by reinforcing their confidence and capability to make meaningful lifestyle adjustments. In this context, ObeCare could be integrated into browsed public health initiatives, using community pharmacies as health promotion hubs, in collaboration with primary care services. This could help to promote lifestyle interventions and facilitate data sharing within existing public health frameworks. Furthermore, these results have significant clinical implications, as they underline the importance of community-based, scalable prevention strategies. Pharmacists can play a pivotal role by identifying risk factors early, referring citizens for further medical assessment, filling informational gaps, and providing ongoing personalized lifestyle counseling.

Our study has a potential limitation. First, data were collected in pharmacies, which may have led to an overrepresentation of individuals already concerned about their health, and approximately 40% of the surveys were collected in Southern Italy, which may influence the national representativeness of the data collected. Despite this, the cohort presented obesity prevalence, sociodemographic, and clinical characteristics similar to those of the overall population, and the validity of the results has been confirmed by the existing literature. Second, the survey implies self-reported data and may be subject to biases (i.e., recall, desirability, and optimistic bias), especially in the theme of obesity (with a high perceived social importance nowadays). To address this issue, the use of BMI and the classification provided by the WHO allows us to minimize wrong underreported weight measures. Furthermore, social conditions, knowledge, and perception information may not be available in the administrative database but only through a survey compilation.

In future works, objective measures of physical activity, such as wearable devices, fitness trackers, or mobile applications, should be employed to obtain more reliable data and reduce biases associated with self-reported measures. Furthermore, a new set of covariates, including ethnicity, socioeconomic, and psychological variables, will be collected.

## 5. Conclusions

The survey conducted provided a clear view of some factors that characterize obesity, its knowledge, and perception as a risk factor among Italian pharmacies’ users. It is undoubtedly essential to raise awareness and implement interventions on this issue, which has a significant impact on public health. A future development of this survey could include administering the questionnaire twice, once before and once after an awareness-raising intervention, to determine its actual impact and isolate the characteristics of the clusters that benefited the most.

## Figures and Tables

**Table 1:** Sociodemographic and clinical characteristics of the respondents overall and according to Body Mass Index (BMI) status.

	Overall	Normal Weight	Overweight	Obese	*p*-Value
		347 (41.76)	286 (34.42)	198 (23.83)	
Sex					
Male	325 (39.11)	91 (28)	149 (45.85)	85 (26.15)	<0.0001
Female	498 (59.93)	252 (50.6)	134 (26.91)	112 (22.49)	
Prefer not to specify	8 (0.96)	4 (50)	3 (37.5)	1 (12.5)	-
Age, median (q1–q3)	48 (35–61)	40 (33–55)	52 (36–64)	56 (43–65)	<0.0001
Employment Status					
Self-employed	132 (15.88)	48 (36.36)	49 (37.12)	35 (26.52)	<0.0001
Employed (physically active)	251 (30.2)	148 (58.96)	71 (28.29)	32 (12.75)	
Employed (sedentary)	191 (22.98)	75 (39.27)	65 (34.03)	51 (26.7)	
Unemployed	34 (4.09)	9 (26.47)	14 (41.18)	11 (32.35)	
Retired	141 (16.97)	34 (24.11)	61 (43.26)	46 (32.62)	
Student	43 (5.17)	23 (53.49)	11 (25.58)	9 (20.93)	
Other	39 (4.69)	10 (25.64)	15 (38.46)	14 (35.9)	
Region					
Nielsen Area 1	149 (17.93)	57 (38.26)	53 (35.57)	39 (26.17)	<0.0001
Nielsen Area 2	121 (14.56)	68 (56.2)	37 (30.58)	16 (13.22)	
Nielsen Area 3	136 (16.37)	57 (41.91)	60 (44.12)	19 (13.97)	
Nielsen Area 4	425 (51.14)	165 (38.82)	136 (32)	124 (29.18)	
Medical Conditions					
No medical condition	382 (45.97)	219 (57.33)	133 (34.82)	30 (7.85)	<0.0001
Diabetes	82 (9.87)	12 (14.63)	27 (32.93)	43 (52.44)	<0.0001
Hypertension	179 (21.54)	27 (15.08)	70 (39.11)	82 (45.81)	<0.0001
Cardiovascular diseases	71 (8.54)	15 (21.13)	29 (40.85)	27 (38.03)	0.0004
Chronic respiratory diseases	31 (3.73)	6 (19.35)	12 (38.71)	13 (41.94)	0.0144
Thyroid disorders	106 (12.76)	45 (42.45)	33 (31.13)	28 (26.42)	0.6919
Gastrointestinal diseases	78 (9.39)	23 (29.49)	29 (37.18)	26 (33.33)	0.0376
Rheumatic diseases	37 (4.45)	9 (24.32)	12 (32.43)	16 (43.24)	0.0112
Autoimmune diseases	25 (3.01)	13 (52)	6 (24)	6 (24)	0.4818
Obesity	74 (8.9)	2 (2.70)	8 (10.81)	64 (86.49)	<0.0001
Oncological diseases	15 (1.81)	4 (26.67)	5 (33.33)	6 (40)	0.2843
Depression or anxiety disorders	50 (6.02)	22 (44)	12 (24)	16 (32)	0.1986
Chronic kidney diseases	5 (0.6)	1 (20)	2 (40)	2 (40)	-
No response	14 (1.68)	8 (57.14)	4 (28.57)	2 (14.29)	-
Lifestyle					
Low	26 (3.13)	5 (19.23)	10 (38.46)	11 (42.31)	<0.0001
Medium-low	297 (35.74)	84 (28.28)	106 (35.69)	107 (36.03)	
Medium-high	413 (49.7)	201 (48.67)	141 (34.14)	71 (17.19)	
High	95 (11.43)	57 (60)	29 (30.53)	9 (9.47)	

## Data Availability

Data may be made available upon reasonable request and subject to confidentiality restrictions.

## Supplementary Materials

The following supporting information can be downloaded at: https://www.mdpi.com/article/10.3390/healthcare13212793/s1.

## Abbreviations

POKQ	Portuguese Obesity Knowledge Questionnaire
HPLP	Health Promoting Lifestyle Profile
BMI	Body Mass Index
WHO	World Health Organization
IQR	Interquartile Range
OR	Odds Ratio
CI	Confidence Interval
