# Supplementary material for: Obesity, Knowledge, and Perceived Risk: Insights from the ObeCare Project Across Italian Territorial Pharmacies
Source: Healthcare (Basel). 2025 Nov 4;13(21):2793. doi: 10.3390/healthcare13212793 (PMC12607364; doi:10.3390/healthcare13212793)
Supplement: Supplementary file 1 [file healthcare-13-02793-s001.zip › Supplementary materials_OBECARE.pdf]

Supplementary materials

Obesity, Knowledge, and Perceived Risk: Insights from the ObeCare Project Across Italian Territorial Pharmacies

S1. Questionnaire Items
General Information

- Pharmacy Name
- Municipality

Section 1. Demographic and Occupational Data

- Sex
  - Male
  - Female
  - Prefer not to say
- Age
- Occupation
  - Unemployed
  - Student
  - Employee (sedentary job)
  - Employee (physically active job)
  - Self-employed
  - Retired
  - Other)
- Weight (kg)
- Height (cm)

Health Conditions

- Have you been diagnosed with one or more of the following conditions? *(Select all that apply)*

  ☐ No diagnosed condition
  ☐ Diabetes
  ☐ Hypertension
  ☐ Cardiovascular diseases (e.g., heart attack, heart failure)
  ☐ Chronic respiratory diseases (e.g., asthma, COPD)
  ☐ Thyroid disorders (e.g., hypothyroidism, hyperthyroidism)
  ☐ Gastrointestinal disorders (e.g., irritable bowel syndrome, Crohn's disease)
  ☐ Chronic kidney disease
  ☐ Rheumatic diseases (e.g., rheumatoid arthritis, osteoporosis)
  ☐ Neurological disorders (e.g., epilepsy, neurodegenerative diseases)
  ☐ Autoimmune diseases (e.g., lupus, multiple sclerosis)
  ☐ Cancer (e.g., tumors, leukemia)
  ☐ Depression or anxiety disorders
  ☐ Prefer not to say

Section 2. Knowledge, Risk Perception about Obesity and Lifestyle Habits

Obesity Knowledge Questions:

- Q1: In Italy, what percentage of adults are affected by excess weight, including both overweight and obesity?
  - 30–40%

    - 40–50%
    - 50–60%
    - Over 60%
    - I don’t know
- Q2: What do you believe are the two main causes of overweight or obesity? *(Select up to two)*
    - ☐ A calorie-rich and nutrient-poor diet
    - ☐ Lack of regular physical activity
    - ☐ Genetic factors
    - ☐ Eating habits learned during childhood
    - ☐ Stress and psychological factors
    - ☐ Use of specific medications
    - ☐ Lack of nutritional knowledge
    - ☐ I don’t know
- Q4: To prevent weight gain, how many calories should a healthy adult who engages in moderate physical activity consume per day?
    - <1200
    - 1201–1500
    - 1501–2500
    - 2501–3500
    - 3501–4500
    - I don’t know
- Q5: In which body area does fat accumulation pose the greatest health risk?
    - Thighs
    - Arms
    - Abdominal region
    - Buttocks
    - I don’t know
- Q6: How is the Body Mass Index (BMI) calculated?
    - Weight ÷ Height
    - Weight ÷ Height^2
    - Height ÷ Weight
    - Height ÷ Weight^2
    - I don’t know
- Q7: A BMI of 22 indicates that a person is...
    - Underweight
    - Normal weight
    - Overweight
    - Obese
    - I don’t know
- Q8: A BMI of 32 indicates that a person is...
    - Normal weight
    - Overweight
    - Obese
    - I don’t know

Risk Perception Questions (Obesity-Related Health Risks):

- Q3: Does regular physical activity reduce abdominal fat accumulation? (1 = Strongly disagree, 5 = Strongly agree)
- Q9: Obesity can lead to type 2 diabetes (1 = No at all likely, 5= Very likely)
- Q10: Obesity can lead to cancer (1 = No at all likely, 5= Very likely)
- Q11: Obesity can cause heart attacks (1 = No at all likely, 5= Very likely)
- Q12: Obesity can lead to high blood pressure (hypertension) (1 = No at all likely, 5= Very likely)
- Q13: Obesity can cause joint pain, such as knee pain (1 = No at all likely, 5= Very likely)

Lifestyle Habits Questions:

- Q14: How often do you engage in at least 30 minutes of physical activity, such as walking, cycling, or jogging? (1 = Never, 5 = Always)
- Q15: How often do you prefer fresh fruits and vegetables over processed foods? (1 = Never, 5 = Always)
- Q16: How often do you limit your intake of sugar and sugary foods? (1 = Never, 5 = Always)
- Q17: How often do you sleep at least 7–8 hours per night to feel rested and refreshed? (1 = Never, 5 = Always)
- Q18: How often do you engage in activities to manage stress, such as meditation, yoga, or hobbies? (1 = Never, 5 = Always)
- Q19: How often do you seek support from family or friends when facing personal difficulties or health issues? (1 = Never, 5 = Always)
- Q20: How often do you undergo health check-ups to monitor your well-being? (1 = Never, 5 = Always)
- Q21: To what extent do you believe your weight affects your overall health? (1 = Not at all, 5= Extremely)
- Q22: How motivated are you to improve your lifestyle (e.g., diet, physical activity)? (1 = Not at all, 5= Extremely)

S2. Questionnaire Feedback Items

Assesses how patients perceived the material and information provided immediately after the education session.

Section 1: Feedback on the material

- How useful did you find the information provided during the education session? (1 = Not at all useful, 5 = Extremely useful)
- Where the explanations provided clear and easy to understand? (Not at all, 5 = Very much so)
- Did the material help you better understand the importance of weight for your health? (1 = Not at all, 5 = Very much so)

Section 2: Motivation to change

- After this session, do you think you will change anything in your lifestyle? (Yes, a lot; Yes, somewhat; No, I don't think so; I don't know)
- How much more motivated do you feel to take care of your health compared to before the session? (1 = Not at all motivated, 5 = Extremely motivated).

Figure S1. Flow-chart of the 1081 collected surveys (Panel A) and geographic distribution of the 831-survey used in the analyses (Panel B).

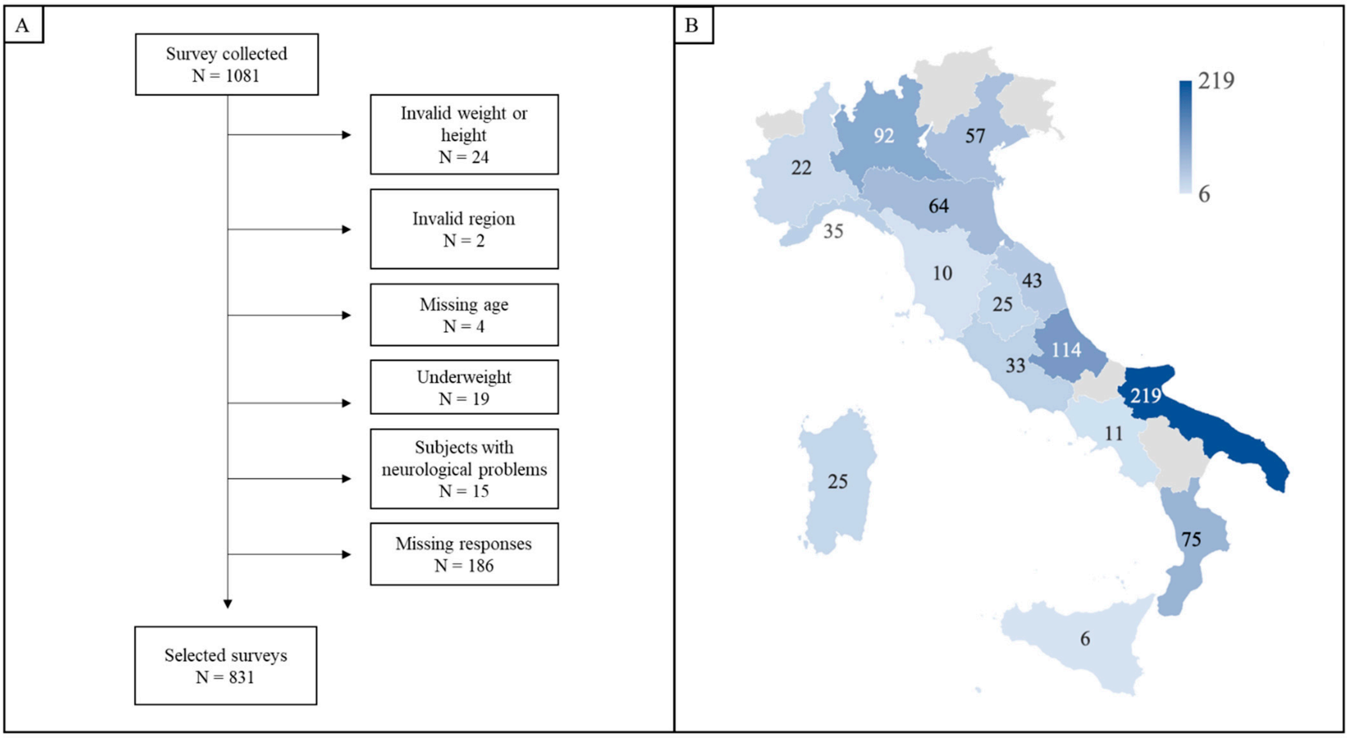

Figure S2. Summary of questionnaire feedback responses. The questionnaire was completed by a smaller number of participants compared with the main one. Extended questions can be found in the Supplementary Materials (S2).

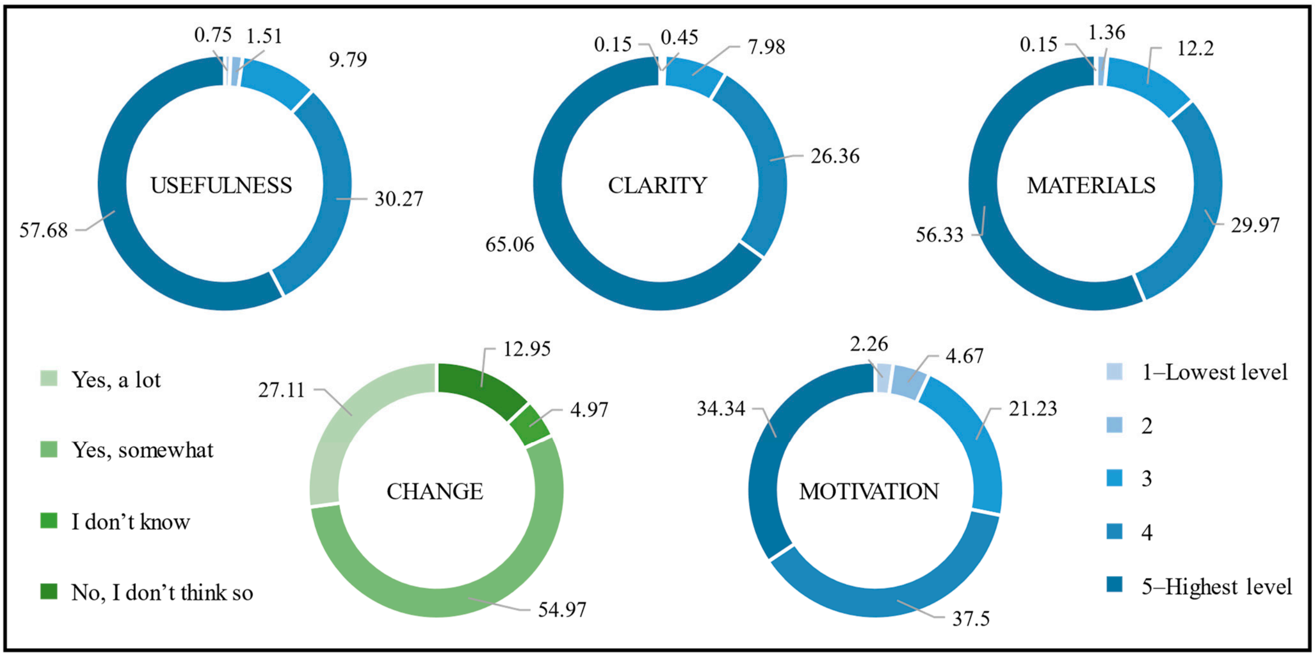

Table S1. Sociodemographic and clinical characteristics of the respondents according to knowledge about obesity and perception about the risks related to obesity levels.

	Knowledge					Perception			
	Low<br>145 (17.45)	Medium-low<br>227 (27.32)	Medium-high<br>305 (36.70)	high<br>154 (18.53)	P-value	Low<br>33 (3.97)	Medium<br>192 (23.10)	High<br>606 (72.92)	P-value
Sex									
Male	49 (15.08)	108 (33.23)	117 (36)	51 (15.69)	0.0137	12 (3.69)	89 (27.38)	224 (68.92)	0.0508
Female	93 (18.67)	117 (23.49)	187 (37.55)	101 (20.28)		19 (3.82)	100 (20.08)	379 (76.1)	
Prefer not to specify	3 (37.5)	2 (25)	1 (12.5)	2 (25)	-	2 (25)	3 (37.5)	3 (37.5)	
Age, median (q1-q3)	55 (38 - 68)	53 (38 - 64)	45 (34 - 58)	40.5 (33 - 55)	<.0001	54 (38 - 65)	51.5 (38 - 65.5)	47.5 (35 - 60)	0.0186
Employment Status									
Self-employed	15 (11.36)	34 (25.76)	57 (43.18)	26 (19.7)	<.0001	5 (3.79)	30 (22.73)	97 (73.48)	0.0007
Employed (physically active)	31 (12.35)	62 (24.7)	104 (41.43)	54 (21.51)		2 (0.8)	40 (15.94)	209 (83.27)	
Employed (sedentary)	26 (13.61)	53 (27.75)	68 (35.6)	44 (23.04)		9 (4.71)	48 (25.13)	134 (70.16)	
Unemployed	7 (20.59)	11 (32.35)	12 (35.29)	4 (11.76)		3 (8.82)	5 (14.71)	26 (76.47)	
Retired	46 (32.62)	51 (36.17)	32 (22.7)	12 (8.51)		7 (4.96)	44 (31.21)	90 (63.83)	
Student	8 (18.6)	5 (11.63)	21 (48.84)	9 (20.93)		3 (6.98)	13 (30.23)	27 (62.79)	
Other	12 (30.77)	11 (28.21)	11 (28.21)	5 (12.82)		4 (10.26)	12 (30.77)	23 (58.97)	
Region									
Nielsen Area 1	32 (21.48)	36 (24.16)	49 (32.89)	32 (21.48)	0.2708	15 (10.07)	28 (18.79)	106 (71.14)	0.0027
Nielsen Area 2	22 (18.18)	26 (21.49)	51 (42.15)	22 (18.18)		3 (2.48)	25 (20.66)	93 (76.86)	
Nielsen Area 3	26 (19.12)	41 (30.15)	52 (38.24)	17 (12.5)		5 (3.68)	32 (23.53)	99 (72.79)	
Nielsen Area 4	65 (15.29)	124 (29.18)	153 (36)	83 (19.53)		10 (2.35)	107 (25.18)	308 (72.47)	
Medical Conditions									
No medical condition	53 (13.87)	92 (24.08)	149 (39.01)	88 (23.04)	0.0008	10 (2.62)	72 (18.85)	300 (78.53)	0.0027
Diabetes	20 (24.39)	26 (31.71)	21 (25.61)	15 (18.29)	0.1019	4 (4.88)	25 (30.49)	53 (64.63)	0.2027
Hypertension	31 (17.32)	65 (36.31)	60 (33.52)	23 (12.85)	0.0099	7 (3.91)	55 (30.73)	117 (65.36)	0.0232
Cardiovascular diseases	14 (19.72)	23 (32.39)	28 (39.44)	6 (8.45)	0.1462	5 (7.04)	21 (29.58)	45 (63.38)	0.1212

Chronic respiratory diseases	5 (16.13)	12 (38.71)	10 (32.26)	4 (12.9)	0.5152	1 (3.23)	8 (25.81)	22 (70.97)	0.9214
Thyroid disorders	20 (18.87)	24 (22.64)	40 (37.74)	22 (20.75)	0.6888	4 (3.77)	21 (19.81)	81 (76.42)	0.6753
Gastrointestinal diseases	15 (19.23)	27 (34.62)	26 (33.33)	10 (12.82)	0.3059	4 (5.13)	18 (23.08)	56 (71.79)	0.8584
Rheumatic diseases	10 (27.03)	10 (27.03)	12 (32.43)	5 (13.51)	0.4308	0 (0)	8 (21.62)	29 (78.38)	0.4205
Autoimmune diseases	2 (8)	9 (36)	11 (44)	3 (12)	-	2 (8)	5 (20)	18 (72)	0.5587
Obesity	13 (17.57)	15 (20.27)	34 (45.95)	12 (16.22)	0.3076	5 (6.76)	15 (20.27)	54 (72.97)	0.3926
Oncological diseases	1 (6.67)	5 (33.33)	6 (40)	3 (20)	-	1 (6.67)	5 (33.33)	9 (60)	-
Depression or anxiety disorders	18 (36)	15 (30)	10 (20)	7 (14)	0.0016	3 (6)	12 (24)	35 (70)	0.7289
Chronic kidney diseases	1 (20)	0 (0)	2 (40)	2 (40)	-	0 (0)	2 (40)	3 (60)	-
No response	2 (14.29)	5 (35.71)	5 (35.71)	2 (14.29)	-	2 (14.29)	9 (64.29)	3 (21.43)	-
BMI_WHO (row perc)									
Normal-Weight	48 (13.83)	85 (24.5)	135 (38.9)	79 (22.77)		9 (2.59)	68 (19.6)	270 (77.81)	
Overweight	49 (17.13)	83 (29.02)	105 (36.71)	49 (17.13)	0.0071	11 (3.85)	69 (24.13)	206 (72.03)	0.0217
Obese	48 (24.24)	59 (29.8)	65 (32.83)	26 (13.13)		13 (6.57)	55 (27.78)	130 (65.66)	
Lifestyle									
Low	13 (50)	2 (7.69)	5 (19.23)	6 (23.08)		6 (23.08)	6 (23.08)	14 (53.85)	
Medium-low	57 (19.19)	81 (27.27)	119 (40.07)	40 (13.47)	<.0001	23 (7.74)	91 (30.64)	183 (61.62)	<.0001
Medium-high	61 (14.77)	110 (26.63)	155 (37.53)	87 (21.07)		4 (0.97)	82 (19.85)	327 (79.18)	
High	14 (14.74)	34 (35.79)	26 (27.37)	21 (22.11)		0 (0)	13 (13.68)	82 (86.32)	
